# Association of prenatal vitamin D concentration with the eruption time of the first primary tooth: A prospective cohort study

**DOI:** 10.34172/joddd.025.41860

**Published:** 2025-06-30

**Authors:** Fahimeh Kooshki, Fatemeh Molaasadolah, Negin Cheraghi

**Affiliations:** ^1^Department of Pediatric Dentistry, School of Dentistry, Shahid Beheshti University of Medical Sciences, Tehran, Iran; ^2^Dental Research Center, Research Institute of Dental Sciences, Shahid Beheshti University of Medical Sciences, Tehran, Iran

**Keywords:** Deciduous tooth, Prenatal care, Tooth eruption, Vitamin D

## Abstract

**Background.:**

The study investigated the association between vitamin D levels during the first and second trimesters of pregnancy and the eruption time of the first primary tooth. Other possible confounders were also assessed, like infants’ birth weight and length and feeding practices. Understanding these factors is crucial for prenatal care strategies to optimize dental development.

**Methods.:**

Our prospective cohort study recruited 120 mother‒infant pairs. The data on maternal plasma levels of vitamin D during the first and second trimesters of pregnancy were obtained from mothers’ medical records. The eruption times of the first primary teeth were recorded during regular oral examinations of the infants. A multiple linear regression model was developed to analyze the relationship between maternal vitamin D levels and the teething time.

**Results.:**

No significant correlation was found between maternal vitamin D levels during the first and second trimesters and the time of the first tooth eruption (*P*=0.594 and *P*=0.722). However, birth weight had a significant inverse relationship (*P*=0.042), indicating that higher birth weight resulted in earlier first tooth eruption.

**Conclusion.:**

The association between maternal vitamin D levels and the time of tooth eruption remains uncertain. However, birth weight is an important determinant of the timing of deciduous tooth eruption, demonstrating a significant inverse relationship. The study’s clinical relevance lies in its contribution to understanding complex prenatal factors that may influence tooth eruption timing, informing healthcare providers about the importance of monitoring birth weight and other potential determinants.

## Introduction

 The eruption of the first primary tooth is one of the most anticipated events of the infant’s development. It is also an important stage because it affects proper alignment and occlusion of permanent dentition.^1^ The onset of the first primary tooth eruption is under strong genetic control; however, some external factors also influence it.^2^ Recently, many researchers have conducted studies to clarify the role of genetic factors, but the role of environmental factors remains unclear.^3^

 Vitamin D has an essential role in phosphate and calcium metabolism. Phosphate and calcium are minerals that affect the mineralization of tooth structures.^4^ Hence, a relationship between plasma levels of vitamin D and initiation of tooth formation can be conjectured. As the first signs of tooth formation are seen at approximately 6‒8 weeks in utero^5^ and vitamin D status in infants is closely related to maternal vitamin D status,^6^ there may be a possible association between maternal vitamin D levels and the eruption time of the first deciduous tooth. Understanding this relationship can inform prenatal care strategies and emphasize overall nutritional support for pregnant women.This approach could ultimately enhance dental outcomes for infants and contribute to better alignment and occlusion of permanent dentition.

 Researchers have found that maternal factors such as childbearing age, infant birth weight, and ethnicity strongly influence tooth eruption.^7-9^ In addition, the nutritional status of mothers, including vitamin D levels, can impact infant development.^10^ It has been demonstrated that infant vitamin D is associated with tooth emergence,^11^ and maternal vitamin D concentration affects the dental development of 10-year-old children.^4^ However, there is a lack of scientific literature regarding the association between maternal vitamin D and the eruption of the first primary tooth, which prompted us to study this issue.

## Methods

###  Study design and setting

 A prospective cohort study was conducted following the principles outlined in the Helsinki Declaration. Parental consent was obtained from all participating mothers. This study was embedded in Zanjan, Iran, among infants of mothers who visited an obstetrician’s private office during their first trimester of pregnancy for prenatal care checkups in August or September 2022.

###  Participants

 The sample size was calculated at 112 subjects, assuming a confidence interval of 95%, power of 90%, and effect size of 0.3 (medium effect size) based on similar previous studies.^4^ Considering the potential for loss to follow-up, 150 mothers and live-birth infants were recruited. Mothers who withdrew from the study (n = 15) and those lost to follow-up (n = 15) were excluded. Thus, our study comprised 120 subjects. The samples were selected using non-random sampling of the mothers visiting a pregnancy care specialist in their first trimester of pregnancy. Infants with congenital birth defects, oral clefts, genetic disorders, systemic diseases, preterm birth, and mothers with systemic illnesses and twin pregnancies were excluded.

###  Data collection

 We obtained parity information, mother’s educational level and age, type of delivery (vaginal or cesarean section), exposure to smoke during pregnancy, feeding practices (bottle feeding/breastfeeding/combination), and infant’s vitamin D supplement use (multivitamin intake/vitamin AD intake/no supplement intake) through interviewing mothers. Mothers’ pre-pregnancy BMI was calculated using the self-reported weights and heights. Mothers were advised to have blood tests in the first trimester (8 ± 2 weeks of gestation) and the second trimester (20 ± 2 weeks of gestation) of pregnancy as part of routine prenatal care checkups advised by obstetricians. These time points were selected because they are critical periods for fetal development and are commonly monitored in prenatal care. Information on maternal vitamin D levels was gathered from mothers’ medical records. 25-hydroxyvitamin D3 levels were measured at the Bahar Laboratory, Zanjan, Iran, in 2022. Vitamin D status was categorized as sufficient ( > 30 ng/mL) and insufficient ( ≤ 30 ng/mL) based on the laboratory’s cut-off values. Gender, birth length, and infant weight were obtained from the records made on the birthday.

###  Follow-up

 After delivery, infants were followed up according to the standard immunization schedule, which included 2, 4, 6, 7, 9,12, and 15 months. Oral cavity examination of the infants was carried out in the vaccination clinic. Vaccinators performed the examinations with the child placed in the mother’s lap, using a mouth mirror and disposable tongue blades under natural light. In this study, any tooth with any part of its crown piercing the gingiva and visible in the oral cavity was considered erupted. The time of eruption of the first tooth was recorded for each child. Additionally, instructions were given to mothers to take frequent observations of their infants’ mouths and record the precise date when the first tooth erupted. Since the infants were examined periodically, tooth eruption would be confirmed at the subsequent examination.

###  Statistical Methods

 The data were analyzed using SPSS 20 (SPSS Inc., IL, USA). Maternal and natal-related confounders were included in a multiple linear regression model with the teething time of the first primary tooth as a dependent variable. All variables except vitamin D levels during the first and second trimesters of pregnancy were adjusted to isolate the effect of maternal vitamin D levels on the time of primary tooth eruption. Vitamin D supplement use and feeding practices were represented by dummy variables, which enabled us to use a single regression equation to describe multiple groups. The significance level was set at 0.05.

## Results

 The study sample comprised 120 children, with 63 females (52.5%) and 57 males (47.5%). The mean age of first tooth appearance was 8.45 months (SD = 2.14), and infants had their lower central incisors erupted. In this study, the onset of the first primary tooth eruption was between 4 and 16 months. Table 1 presents the statistical indices of qualitative values.

**Table 1:** Characteristics of mothers and infants included in the study

**Maternal/natal characteristics**	**N**	**Minimum**	**Maximum**	**Mean**	**Standard deviation**
Maternal age	120	18	43	30.01	5.30
Prepregnancy BMI	120	16	35	25.38	3.67
Birth length of infant	115	42	57	49.66	2.16
Birth weight of infant	120	2	5	3.44	.53
Eruption time of first primary tooth	120	4	16	8.45	2.14

 The mean age of tooth eruption in infants of mothers with sufficient vitamin D levels ( > 30 ng/mL) during the first trimester of pregnancy was 8.36 months, while the mean age of tooth eruption in infants of mothers with insufficient vitamin D levels ( ≤ 30 ng/mL) during the first trimester was 8.54 months. During the second trimester of pregnancy, infants of mothers who had sufficient vitamin D levels erupted their first teeth at an average age of 8.51 months, whereas infants of mothers who had insufficient vitamin D levels erupted their first teeth at an average age of 8.44 months (Table 2).

**Table 2:** Statistical indices of eruption time of the first primary tooth in infants considering qualitative variables

**Factor**	**Levels**	**Mean**	**N**	**Standard deviation**
Maternal vitamin D level in first trimester^a^	Insufficient ( ≤ 30 ng/mL)	8.54	63	2.19
	Sufficient ( > 30 ng/mL)	8.36	56	2.11
Maternal vitamin D level in second trimester^b^	Insufficient ( ≤ 30 ng/mL)	8.44	75	2.27
	Sufficient ( > 30 ng/mL)	8.51	39	2.00
Parity	1	8.06	50	2.12
	2	8.68	41	1.88
	3	9.13	23	2.45
	4	7.50	6	2.17
Education level	Primary school education	10.40	5	1.34
	Diploma	8.24	34	2.06
	Associate degree	8.17	6	1.83
	Bachelor’s degree	8.33	63	2.05
	Master’s degree	9.00	12	2.92
Gender	Female	8.70	63	2.11
	Male	8.18	57	2.15
Type of delivery	Caesarean section	8.45	94	2.30
	Normal delivery	8.46	26	1.48
Feeding practice	Bottle feeding	8.33	18	1.75
	Breastfeeding	8.70	73	2.14
	Combination	7.90	29	2.29
Vitamin D supplement use	Vitamin AD intake	8.45	98	2.23
	No supplement intake	8.00	6	2.10
	Multivitamin intake	8.63	16	1.59
Exposure to smoke	No	8.55	106	2.17
	Yes	7.71	14	1.77

^a^ Missing data for one participant. ^b^ Missing data for 6 participants.

 Instead of categorizing vitamin D levels based on clinical cut-offs, we analyzed them continuously to obtain more accurate results. The relationship between maternal 25(OH)D concentrations during the first trimester of pregnancy and the timing of the eruption of the first primary teeth was assessed, and a regression coefficient of -0.009 was achieved, implying a reverse correlation. However, this relationship was not statistically significant (*P* = 0.594). The correlation between vitamin D levels during the second trimester and the eruption timing of the first primary teeth was also investigated. According to the negative regression coefficient (β = -0.008) and the *P* value of 0.722, these variables were inversely correlated with no statistical significance. This indicates that higher levels of maternal 25(OH)D may accelerate the eruption of primary teeth, but no statistically significant association was observed (Table 3).

**Table 3:** Results of multiple linear regression analysis after adjustment of all variables except vitamin D levels during the first and second trimesters of pregnancy

		**Unstandardized coefficients**		**Standardized coefficients**	**t**	**Sig.**
		**B**	**Standard error**	**Beta**		
(Constant)		13.590	5.546		2.450	0.016
Parity		0.086	0.301	0.035	0.286	0.776
Education level		-0.046	0.216	-0.023	-0.212	0.833
Maternal age		0.020	0.049	0.047	0.410	0.683
Prepregnancy BMI		0.044	0.065	0.074	0.676	0.501
Type of delivery		-0.093	0.553	-0.018	-0.169	0.866
Birth length of infant		-0.056	0.105	-0.056	-0.527	0.599
Birth weight of infant		-0.886	0.429	-0.219	-2.064	0.042
Exposure to smoke		-0.901	0.713	-0.136	-1.264	0.209
Maternal vitamin D level in first trimester		-0.009	0.017	-0.054	-.535	0.594
Maternal vitamin D level in second trimester		-0.008	0.021	-0.039	-0.357	0.722
Vitamin D supplement use	Vitamin AD intake. dummy	1.304	1.067	.230	1.222	0.225
	Multivitamin intake. dummy	1.262	1.197	.196	1.055	0.294
Feeding practice	Breastfeeding. dummy	0.358	0.670	0.080	0.533	0.595
	Combination. dummy	-0.314	0.750	-0.061	-.419	0.676
Gender		-0.585	0.447	-0.135	-1.309	0.194

 According to the results of multiple linear regression analysis (Table 3), the birth weights of the infants were the only variable among those listed in Table 3 to have a significant effect on the eruption of the first primary tooth (*P* = 0.042), with an inverse relationship (β = -0.886), suggesting that heavier infants were more likely to have their first primary tooth erupted earlier.

## Discussion

 This study assessed the correlation between prenatal vitamin D levels and the eruption time of the first deciduous tooth. Our results indicated that maternal vitamin D concentrations in both the first and second trimesters were inversely correlated with the age of the first primary tooth emergence; however, the relationship was not statistically significant. The mean age of tooth eruption was 8.36 months for infants of mothers with sufficient vitamin D levels in the first trimester and 8.54 months for those with insufficient levels, with a *P* value of 0.594. In the second trimester, the mean age was 8.51 months for sufficient vitamin D levels and 8.44 months for insufficient levels, with a *P *value of 0.722. The only statistically significant predictor of the time of the first tooth eruption was the infant’s birth weight, with an inverse correlation. This means low birth weight in infants results in delayed primary tooth eruption.

 The inverse association between prenatal vitamin D levels and the time of the first primary tooth eruption (with regression coefficients of -0.009 and -0.008 for the first and second trimesters, respectively) indicates that higher levels of 25(OH)D may be correlated with earlier first tooth eruption. However, this relationship was not statistically significant, indicating that while vitamin D plays an important role in overall dental and skeletal health,^12^ its impact on the eruption time of the teeth may be limited.

 Dhamo et al^4^ investigated the association between mid-pregnancy vitamin D concentration and the dental development of 10-year-old children. The results of their study implied that higher maternal 25(OH)D concentrations are related to decelerated dental development in childhood. The inverse correlation between maternal vitamin D levels during pregnancy and infants’ teething time aligns with their findings, suggesting that higher plasma vitamin D levels may accelerate tooth eruption and result in lower dental age.

 Regarding the results of other studies in this field, tooth eruption time is affected by both genetic and exogenic factors.^13^ While adequate vitamin D levels during pregnancy influence many aspects of infant development,^14^ its role in influencing the time of primary tooth eruption seems less noticeable than other factors, such as genetic susceptibility. Many studies have been performed to identify genes affecting dental maturity, indicating that dental development is a complex polygenic process.^15,16^ Studies concerning primary failure of eruption also represent the significant role of genetic mutations, emphasizing that tooth eruption is predominantly governed by genetic factors.^13,17-20^ Therefore, the role of an individual’s genetic makeup in the timing and sequence of primary teeth eruption should not be underestimated. Clinically, this implies that while ensuring sufficient maternal vitamin D levels is essential for general health, other factors may be more important when addressing concerns about delayed tooth eruption.

 The relationship between infants’ birth weights and the time of their first primary tooth eruption has been evaluated in several studies. In 2019, Wu et al. found that children with low birth weight (< 2500 g) experienced delayed tooth eruption. Similarly, other studies showed that teething time is inversely correlated with the birth weight of infants.^21,22^ According to a study by Merglova et al,^23^ one-year-old infants with very low and extremely low birth weight had fewer erupted primary teeth and a delayed eruption time for their first teeth. Our research also pointed to the statistically significant effect of the infants’ birth weight on teething time (*P* = 0.042), corroborating other studies’ findings. Birth weight appears to be a strong determinant of the timing of tooth eruption. Low birth weight often indicates poor nutrition during pregnancy, suggesting that adequate nutrition may accelerate tooth eruption.

 In 2016, Un Lam et al^24^ proposed that the rate of weight gain in infants during the first three months of life significantly affects the first tooth eruption time. As the crown mineralization of primary mandibular central incisors continues until the first three months of infancy,^25^ nutritional status and weight gain during this period may have an important effect on teething time. Hence, including this confounder in our study would have been better.

 To the best of our knowledge, this is the first study to explore the correlation between maternal vitamin D levels and the timing of primary tooth eruption. Although this relationship was not statistically significant, the study was designed and conducted using rigorous methods. We considered and adjusted for several potential confounders, such as maternal age, pre-pregnancy BMI, birth weight, birth length, feeding practices, and vitamin D supplement use, which is one of the strengths of our study. By adjusting these variables, we wanted to focus specifically on the influence of maternal vitamin D on teething chronology. Also, the results of this study help improve our understanding of how birth weight, as an indicator of prenatal nutritional status, might affect infant development.

 A limitation of the present study was the small number of infants over 12 months of age at eruption (only 4 cases), making it difficult to assess a sufficient number of them. This might not align with recent trends in tooth eruption timing, which could affect the generalizability of our findings. Also, potential causal factors such as genetic factors, other nutritional deficiencies, sunlight exposure, and weight gain during the first three months of infancy were not included in this study, which might have influenced the results. Future studies should investigate how genetics and vitamin D levels after birth impact tooth development. This could help clarify how factors before and after birth interact to influence tooth eruption timing. Longitudinal studies with larger sample sizes and more diverse populations are recommended for future research to help understand the interactions between these factors and tooth eruption timing.

## Conclusion

 In conclusion, it is difficult to confirm the correlation between maternal vitamin D concentrations and the eruption time of the first tooth. Birth weight, however, emerged as an important determinant of tooth eruption timing.

## Competing Interests

 The authors declare that they have no conflicts of interest to disclose.

## Ethical Approval

 This prospective cohort study was conducted with the approval of the Shahid Beheshti University of Medical Sciences Ethics Committee (Approval Number: IR.SBMU.DRC.REC.1402.023). Written informed consent was obtained from the mothers of all participating infants before their involvement in the study.
